# ITGB5 promotes innate radiation resistance in pancreatic adenocarcinoma by promoting DNA damage repair and the MEK/ERK signaling pathway

**DOI:** 10.3389/fonc.2022.887068

**Published:** 2022-09-30

**Authors:** Xin Wen, Si Chen, Xueting Chen, Hui Qiu, Wei Wang, Nie Zhang, Wanming Liu, Tingting Wang, Xin Ding, Longzhen Zhang

**Affiliations:** ^1^ Department of Radiation Oncology, Affiliated Hospital of Xuzhou Medical University, Xuzhou, China; ^2^ Cancer Institute of Xuzhou Medical University, Xuzhou, China; ^3^ Department of Radiation Oncology, The Second Affiliated Hospital of Xuzhou Medical University, Xuzhou, China; ^4^ Jiangsu Center for the Collaboration and Innovation of Cancer Biotherapy, Xuzhou, China

**Keywords:** pancreatic adenocarcinoma (PAAD), ITGB5, radio-sensitivity, MEK/ERK signaling pathway, DNA damage repair

## Abstract

Pancreatic adenocarcinoma (PAAD) is one of the most aggressive digestive system tumors in the world, with a low early diagnosis rate and a high mortality. Integrin beta 5 (ITGB5) is demonstrated to be a potent tumor promoter in several carcinomas. However, it is unknown whether ITGB5 participates in the occurrence and development of PAAD. In this study, we confirmed a high expression of ITGB5 in PAAD and its role in promoting invasiveness and transitivity in PAAD. Besides, the knockdown of ITGB5 increased cell sensitivity to radiation by promoting DNA damage repair and the MEK/ERK signaling pathway. Collectively, these results show that ITGB5 plays an essential role in pancreatic cancer growth and survival.

## Introduction

Pancreatic adenocarcinoma (PAAD), also known as “the king of carcinoma”, is one of the most aggressive cancers. In recent decades, in the context of other cancer treatment outcomes significantly improved, it has remained a huge clinical challenge to manage in PAAD. Due to the lower early diagnosis and a lack of effective therapeutic options, the 5-year survival rate of PAAD is only 9% (1, 2). According to changing demographics and the annual average percentage change in incidence rate and death rate, by 2030, PAAD is projected to be the second leading cause of cancer-related death (3). It may be one effective way to improve the current poor treatment status by finding PAAD surface molecules that can be targeted by therapeutics.

Integrins are a superfamily of heterodimeric somatic surface receptors and are composed of α (120–185 kD) and β (90–110 kD) subunits. So far, at least 18 α subunits and 8 β subunits have been found, resulting in 24 heterodimers (4). Multiple integrins have been demonstrated in tumor progression by regulating different biological functions, making them attractive and potential target molecules for tumor therapy (5–7). Integrin-β5 (ITGB5) is a member of integrins and was proved to be associated with pathological processes in several tumors (6, 8, 9). However, the function of ITGB5 in PAAD is not clear.

On the one hand, radiotherapy (RT) is one effective antitumor technology for local control in advanced PAAD (10, 11). On the other hand, irradiation can induce DNA damage response, also known as DDR, which may protect cells from irradiation-induced DNA damage, ultimately showing radio-resistance (12). RT has limited application because of innate radiotherapy resistance in PAAD. Currently, gemcitabine and capecitabine were reported as radiosensitizers for PAAD, but the overt toxicity and side effects are usually intolerant. ITGB5 was reported to contribute to chemoresistance such as cisplatin in numerous malignancies (13, 14). However, whether ITGB5 expression can weaken or strengthen innate resistance to radiotherapy in PAAD is unknown.

Studies have reported that mitogen-activated protein kinase (MAPK)/extracellular signal-regulated kinase (ERK) pathways took part in the regulation of cell proliferation by transferring signals from the cell membrane to the nucleus. In the MAPK/ERK pathway, ligand binding to the plasma membrane receptor tyrosine kinases results in small GTPase RAS activation, which is followed by a series of protein interactions and phosphorylation events such as RAF phosphorylation and MAPK kinase (MEK) activation. ERK is the only substrate of MEK and is phosphorylated by MEK directly (15, 16). ERK phosphorylation activates multiple substrates, which then regulate cell proliferation, apoptosis, metabolism, and immune response (17). The deregulation of the MEK/ERK pathway is common in cancer, such as ovarian cancer, melanoma, and PAAD (17, 18). Besides, MEK/ERK inhibitors are proved to increase the radiosensitivity of cancer cells (19). However, the relationship of ITGB5 and MEK/ERK pathway is unknown.

**Figure 1:**
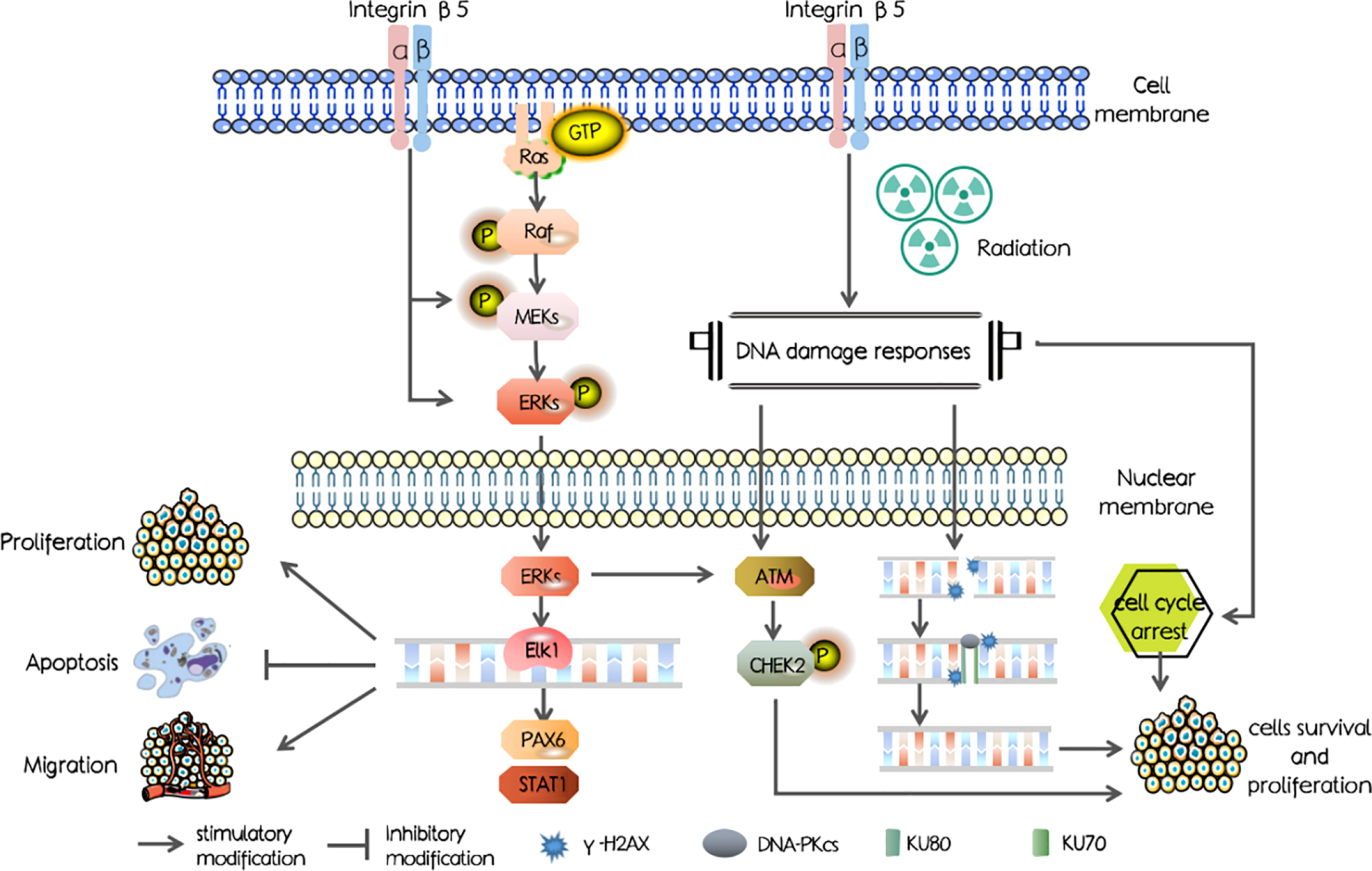
The schematic diagram of mechanisms in our study.

In our study, we first investigated the ITGB5 expression level in PAAD and abnormally elevated ITGB5 contributed to a poor survival outcome. Mechanisms shown in **Figure 1** were examined and showed that ITGB5 promotes proliferation, invasion, and migration by activating the MEK/ERK pathway; besides, ITGB5 protected pancreatic cancer cells from radiation through activating the DNA injury repair pathway including DNA-PK as well as ATM.

## Materials and methods

### Cell culture

Human pancreatic cancer cell lines including BXPC3, PANC-1, and ASPC-1 as well as human normal pancreatic cells HPDE6-C7 were obtained from the Jiangsu Center for the Collaboration and Innovation of Cancer Biotherapy. PANC-1, ASPC-1, and HPDE6-C7 were cultured in DMEM (sigma, D5796) with 10% fetal bovine serum (FBS), and 1% penicillin, streptomycin, and BXPC3 were cultured in RPMI-1640 medium (sigma, R8758) with 10% FBS and 1% penicillin and streptomycin at 37°C, 5% CO_2_.

### Transfection

We designed two small guide RNAs for knocking out ITGB5 by the CHOPCHOP web tool (20):

sgITGB5-1: 5′-CACCGCTTTCTCCTACACGGCACCG-3′

sgITGB5-2: 5′-CACCGCTGGCGAACCTGTAGCTGGA-3′

293T cells growing on a 6-cm dish were firstly transfected with 4 μg pCDH-ITGB5 or Lenticrispr-ITGB5 or control vector (pCDH, Lenticrispr V2), 3 μg PSPAX2, 1 μg PMD2G, and Lipofectamine 2000 for obtaining lentivirus expressing ITGB5. Forty-eight hours after transfection, the culture medium supernatant was centrifuged to remove cell debris at 1,000 rpm, 5 min for infection. PANC1 or BXPC3 cells seeded on a 6-cm dish with 60% cell density were infected by the above supernatant, and then stable ITGB5 knockout cell lines (ITGB5-KO) or ITGB5 overexpression cell lines (ITGB5-OE) with puromycin for 3 days were selected. Western blot analysis was used to evaluate the ITGB5 expression efficiency.

### Antibodies

ITGB5 was obtained from Abcam (ab184312, Abcam) and Novus Biologicals (NBP1-88117, Novus Biologicals) for Western blot and immunohistochemistry (IHC) studies, respectively. MEK1/2 (9154T) and γ-H_2_AX (9718S) were from Cell Signaling Technology (CST). Ki67 (A11005), ERK, and pERK (A11116) were from ABclonal. KU70 (10723-1-AP), KU80 (16389-1-AP), RAD51 (14961-1-AP), ATM (27156-1-AP), CHEK2 (13954-1-AP), Phospho-CHEK2 (Thr68) (29012-1-AP), PAX6 (12323-1-AP), STAT1 (10144-2-AP), GAPDH (10494-1-AP), and β-actin (23660-1-AP) were from Proteintech. DNA-PKcs (BS1092) was from Bioworld. HRP-Goat Anti-Rabbit IgG (VA001) and HRP-Goat Anti-Mouse IgG (VA002) were obtained from Vicmed.

### Immunohistochemistry

IHC was conducted as previously described (21). Simply, we used the PV-9000 two-step immunohistochemistry kit and DAB color reagent (ZSBIO) to detect antigens in human PAAD tissue microarrays (TMA) (HPanA 150Su01, Shanghai Outdo Biotech, China) and mouse tissue samples according to the manufacturer’s instruction.

### Cell proliferation and colony formation assay

CCK-8 and colony formation assays were used to evaluate the influence of ITGB5 on the tumor cells’ growth rate. ITGB5-KO, ITGB5-OE, and control BXPC3 and PANC-1 cells were seeded into 96-well plates (1,000 cells per well). The changes in cell number were detected with a CCK-8 assay kit continuously for 4 days. The absorbance at 450 nm (OD_450_) of cells was measured by a microplate reader. Furthermore, cell proliferation ability was further detected by colony formation assays. The equal number of cancer cells was seeded in six-well plates with appropriate density and cultured for 10–14 days at 37°C, 5% CO_2_. The medium was replaced every 3 days. In radiation sensitization assays, cancer cells with appropriate cell intensity seeded in six-well plates were one-off irradiated with 0, 2, 4, 6, and 8 Gy, respectively, at a dose rate of 300 cGy/min. All cells were irradiated with 6-MeV X-ray, and the source-surface distance was 100 cm. Besides, the surface of plates was covered with a 1-cm-thick tissue equivalent filler. After radiation, cells were cultured until the visible colonies formed about 10~14 days later. Then, cell clones were fixed with 4% paraformaldehyde (Vicmed, China) and stained with crystal violet (Beyotime, China). The number of colonies was counted by ImageJ software. Plating efficiency (PE) was calculated by the following formula: (the number of colonies/the number of cells plated) × 100%. Survival fraction (SF) was calculated by the following formula: (PE of experimental group/PE of control group) × 100% (22, 23). The relevant parameters such as α, β, D0, Dq, and sensitization enhancement ratio (SER_D0_ = D0 of experimental group/D0 of control group) and surviving curves were obtained according to the single-hit multitarget model (SF = 1-(1-e^-D/D0^)^N^, Dq = D0 × lnN) and linear–quadratic model (SF = e^-αD-βD2^) by GraphPad Prism 8.0 software. Each experiment was repeated three times.

### Transwell assay

Transwell assay was used to assess the cell invasion and migration ability. The cell density was adjusted to 2.5 × 10^5^ cells/ml in serum-free medium, and 200 µl was added to the upper chamber with or without Matrigel. Eight hundred microliters of medium with 20% FBS was added to the lower chamber. Cells on the membrane filters were fixed and stained with crystal violet. The number of invasive cells was photographed using a bright microscope and then calculated. Each experiment was repeated three times.

### Western blot

Western blot (WB) was used to evaluate the effect of ITGB5 knockout or overexpression efficiency and irradiation (IR) on the expression of DNA damage proteins or other signal pathway proteins in cancer cells with different ITGB5 expression levels. For evaluating expression efficiency, the adherent cancer cells seeded in six-well plates were directly harvested for WB; for expression detection of other proteins, the adherent cancer cells seeded in six-well plates were irradiated with 6 Gy at a dose rate of 300 cGy/min. At different time points after irradiation, cells were harvested for WB as in a previous study (22). Briefly, membranes were incubated with primary antibodies at 4°C overnight and with secondary antibodies for 1 h at room temperature. Then, membranes were washed three times and then imaged with Chemiluminescence Image System. The band intensity was analyzed by ImageJ software. Each experiment was repeated three times.

### Cell-cycle assay

Cell-cycle assay was used to examine the effect of ITGB5 expression on pancreatic cancer cell-cycle distribution. Briefly, cancer cells (ITGB5-KO, ITGB-OE, and control BXPC3 cells) were seeded in 6-cm plates with appropriate density. All cells were irradiated with 6 Gy at dose rate of 300 cGy/min. Twenty-four hours after irradiation, all cells were directly harvested using a cell cycle assay kit (KeyGEN BioTECH, KGA512) according to vendors’ instructions and analyzed by flow cytometry. Each experiment was repeated three times.

### Xenograft assay

All experiments *in vivo* were conducted ethically on the basis of the Jiangsu Council on Animal Care. Four- to 6-week-old male BALB/c nude mice (GemPharmatech, China) were adaptively reared at the SPF level Laboratory Animal Center for 1 week before the experiment began. The distal right or left lower extremities of mice were subcutaneously injected with 5 × 10^6^ BXPC3 or PANC-1 cells suspended in 200 μl medium without FBS. Tumor volumes were measured with a caliper every 2 days and calculated by the following formulation: V = 0.5 × a × b^2^ (a is the larger diameter and b is the smaller diameter). Thirty days after subcutaneous neoplasia, mice were euthanized by overdose of carbon dioxide. Subcutaneous tumors were dissected completely and weighted, which were then processed for further detection of ITGB5 and Ki67 expression by IHC. Tumor growth curves were obtained by GraphPad Prism 8.0 software.

### Bioinformatics analyses

The expression level of ITGB5 in different types of tumors was obtained from the GEPIA website (http://gepia.cancerpku.cn/) (24). Gene expression data of PAAD were downloaded and processed from TCGA-PAAD (https://portal.gdc.cancer.gov/) and GTEx (https://xenabrowser.net/datapages/) databases as in our previous study (25). Simply, a total of 171 normal pancreatic samples (167 from GTEx and four from TCGA) and 178 PAAD samples were used to analyze the ITGB5 expression difference. According to the clinical information downloaded from TCGA-PAAD, the samples with incomplete follow-up information were deleted and, ultimately, 173 PAAD samples were used for survival analysis and clinical correlation analysis. Kaplan–Meier (KM) survival curves were used to evaluate the influence of ITGB5 on overall survival (OS) by the “survminer” package in R (V.4.0.2) software. The cutoff value of ITGB5 expression was its median value. Wilcox test was used for clinical correlation analysis of ITGB5 expression in R (V.4.0.2) software.

### Statistical analysis

All statistical analyses were conducted by SPSS 25. Data were presented as means ± standard deviation (SD). The statistical significance difference was compared by two-sample t-test or one-way ANOVA. The *X* ^2^ test was done to analyze the association between ITGB5 expression and clinical features of PAAD. Kaplan–Meier (K-M) analysis was used to assess survival curves, and log-rank test was used to compare survival curves among groups. p < 0.05 was considered significant.

## Results

### ITGB5 overexpression related with worse survival in PAAD patients

**Figure 2:**
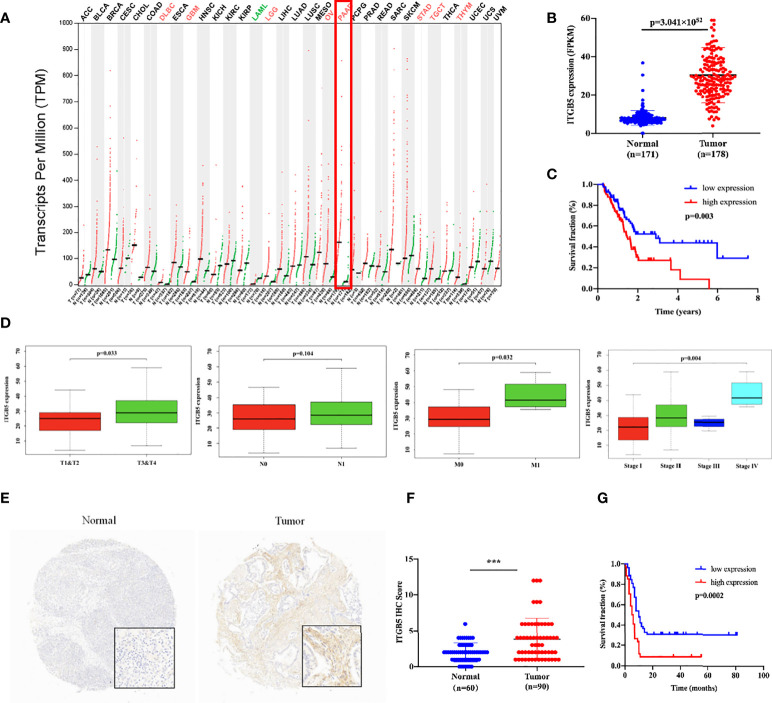
ITGB5 overexpression is related with worse prognosis in PAAD patients. **(A)** ITGB5 expression in various cancer types based on the online GEPIA database. **(B)** ITGB5 expression is elevated in PAAD based on TCGA and GTEx databases. **(C)** Upregulated ITGB5 was associated with worse prognosis based on TCGA database. **(D)** The clinical relevance of ITGB5 expression in PAAD analyzed by TCGA database (T staging, N staging, M staging, and stage, respectively, from left to right). **(E)** Representative images of IHC on PAAD TMA. **(F)** ITGB5 expression scores in TMA. **(G)** The survival curve by TMA using Kaplan–Meier survival analyses. ***p < 0.001.

Firstly, we analyzed ITGB5 expression in cancers by the GEPIA database (**Figure 2A**). ITGB5 is upregulated in numerous cancer types including PAAD (N_tumor_ = 179, N_normal_ = 171) compared to normal samples. Then, we analyzed the ITGB5 expression difference between PAAD samples and normal pancreatic samples based on TCGA and GTEx databases; ITGB5 is significantly upregulated in PAAD (**Figure 2B**). KM survival analysis revealed that ITGB5 overexpression is highly bound up with poor prognosis (**Figure 2C**). Concerning the clinical correlation (**Figure 2D**), ITGB5 expression was clearly upregulated in patients with a T3&T4 stage or distant metastases compared to patients with T1&T2 stage or non-distant metastases, which highlighted the invasive and metastatic potential of ITGB5 expression in pancreatic cancer. Lastly, we examined ITGB5 protein expression in TMA containing 90 PAAD samples and 60 normal pancreatic samples. As shown in **Figures 2E, F**, consistent with the results from the database analysis, ITGB5 is highly regulated in PAAD samples. We further analyzed the relationship of ITGB5 with prognosis and clinical characteristics in PAAD. Samples with incomplete clinical factors were rejected, and a total of 87 PAAD samples were included in the analysis. Clinical characteristics of 90 PAAD samples are summarized in **Table 1**, of which 39 samples were 60 years old or younger and 48 samples were 60 years old or older. Fifty-six were men and 31 were women. Concerning pancreatic cancer infiltration, 31 samples were coupled with perineural invasion, 52 samples were coupled with lymph node metastasis, and 50 samples were located in the head of pancreas. Most of the PAAD samples had earlier clinical stages, 27 samples were stage I, 59 samples were stage II, and the rest of the samples were stage IV. ITGB5 expression was related with tumor size, lymph node metastasis, and clinical stage significantly (p < 0.05). Besides this, ITGB5 overexpression in PAAD predicted unsatisfactory prognosis (**Figure 2G**). In conclusion, ITGB5 is not only highly regulated in PAAD compared to normal pancreas but also related with poor prognosis, which can be a potential prognostic marker in PAAD.

**Table 1:** The relationship between ITGB5 expression and clinical characteristics in PAAD.

Variable	n	ITGB5 expression		*P*-value
		High	Low	
Age				0.583
≤60	39	14	25	
>60	48	20	28	
Gender				0.958
Male	56	22	34	
Female	31	12	19	
Tumor size				0.001
≤4	45	10	35	
>4	42	24	18	
Perineural invasion				0.075
Negative	56	18	28	
Positive	31	16	15	
Lymph node metastasis				0.041
Negative	35	6	29	
Positive	52	28	24	
TNM stage				0.005
I	27	4	23	
II	59	29	30	
IIÌ	0	0	0	
IV	1	1	0	
Tumor location				0.810
Head	50	19	31	
Non-head	37	15	22	

### ITGB5 promotes tumor growth, invasion, and metastasis

**Figure 3:**
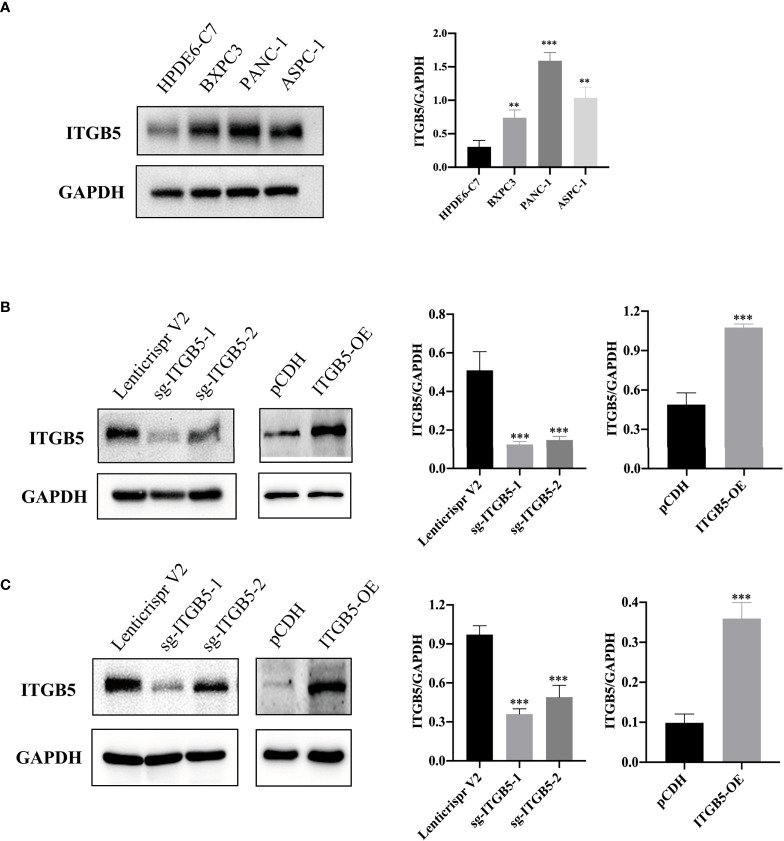
ITGB5 expression in pancreatic cancer cells by Western blot. **(A)** ITGB5 expression in normal pancreatic cell (HPDE6-C7) and pancreatic cancer cells (BXPC3, PANC-1, ASPC1). **(B)** verification of ITGB5 knockout, ITGB5 overexpression, and control PANC-1 cells. **(C)** Verification of ITGB5 knockout, ITGB5 overexpression, and control BXPC3 cells. **p < 0.01, ***p < 0.001.

**Figure 4:**
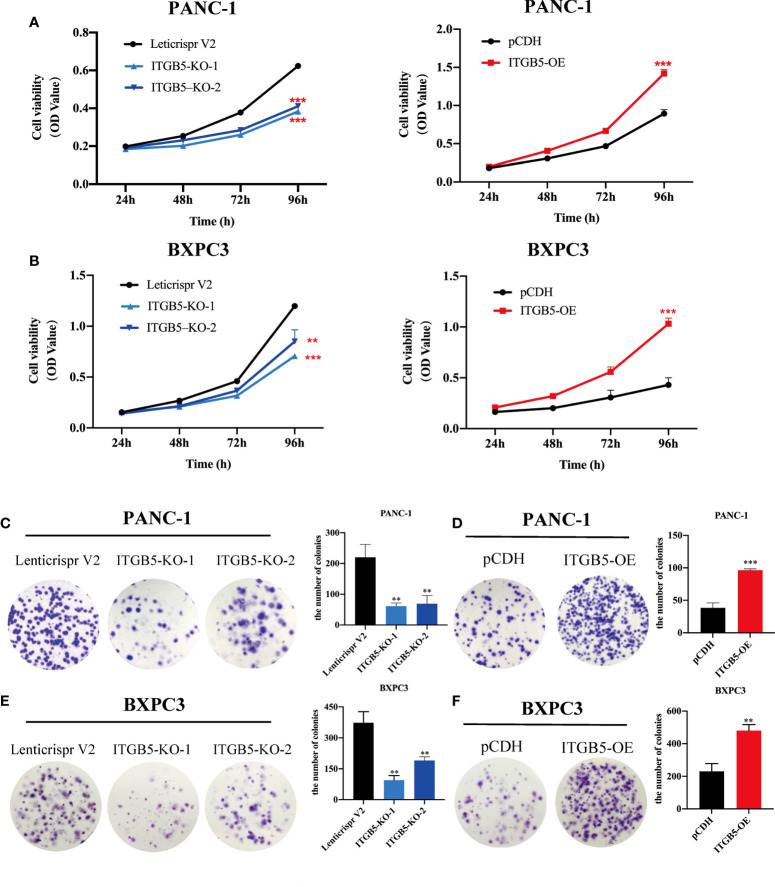
The effect of ITGB5 on proliferation of pancreatic cancer cells *in vitro* by CCK8 **(A, B)** and colony formation assay **(C-F)**. **p < 0.01, ***p < 0.001.

**Figure 5:**
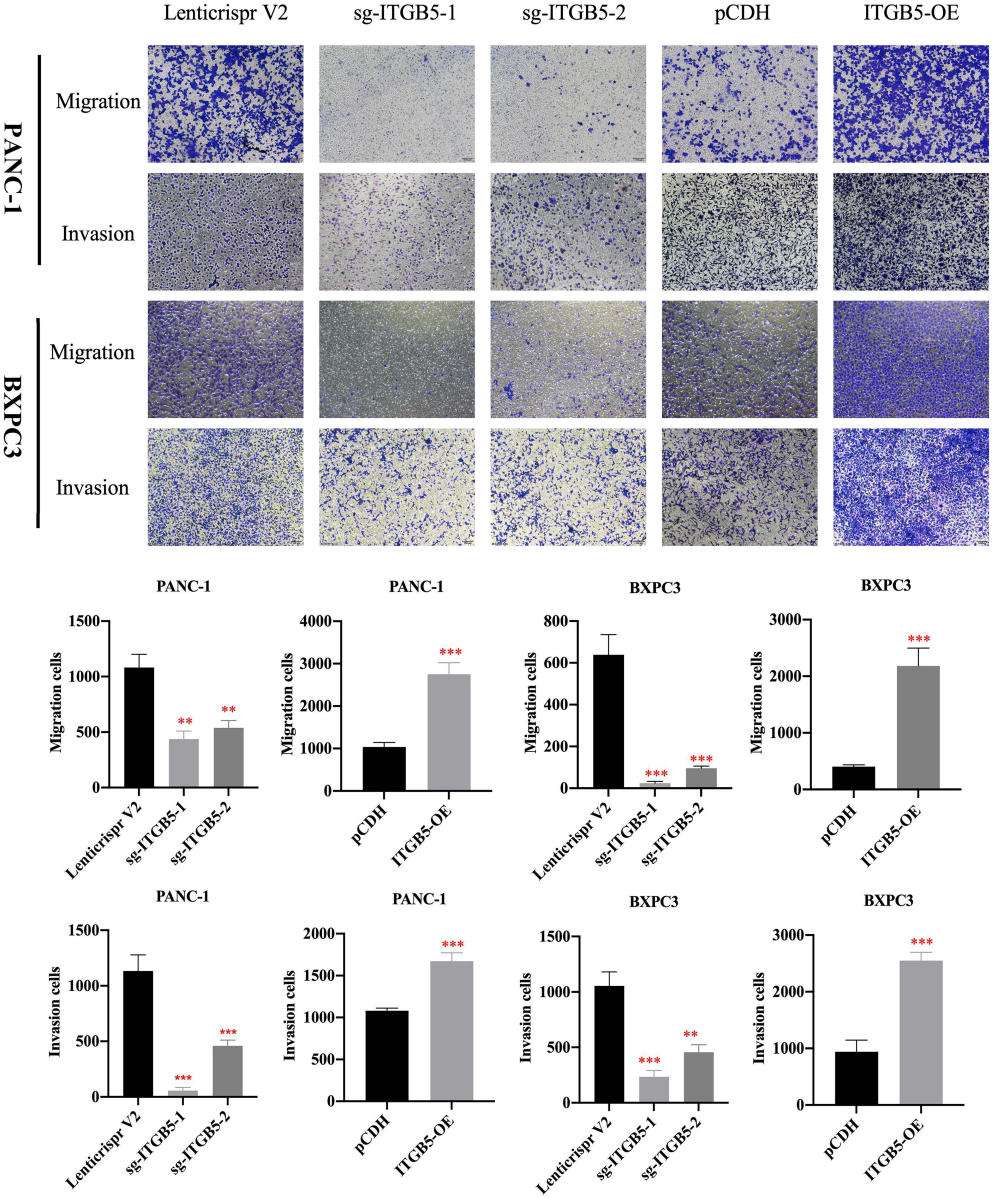
The effect of ITGB5 expression on migration and invasion on pancreatic cancer cells. **p<0.01, ***p<0.001.

To investigate the role of ITGB5 in PAAD, we established ITGB5 knockout or overexpression pancreatic cancer cell lines. Firstly, we examined the expression of ITGB5 in three pancreatic cancer cell lines (BXPC3, PANC-1, and ASPC1) and one normal pancreatic cell line (HPDE6-C7). As shown in **Figure 3A**, compared to HPDE6-C7, ITGB5 expression was obviously upregulated in BXPC3, PANC-1, and ASPC1. Considering that the expression of ITGB5 was highest in PANC-1 and lowest in BXPC3, we used PANC-1 and BXPC3 to construct stable ITGB5 knockout or overexpression cells (**Figures 3B, C**). CCK-8 results showed that compared to control cells (Lenticrispr V2 or pCDH), knocking out ITGB5 expression in PANC-1 or BXPC3 (ITGB5-KO-1, ITGB5-KO-2) obviously inhibited cell proliferation; on the contrary, upregulated ITGB5 expression in PANC-1 or BXPC3 (ITGB5-OE) obviously promoted cell proliferation (**Figures 4A, B**). Colony formation assays reached the same conclusion (**Figures 4C-F**). Transwell assay showed that ITGB5 overexpression promoted migration and invasion of PAAD cells, which was oppositely inhibited by the silence of ITGB5 (**Figure 5**).

### ITGB5 knockout inhibits xenograft tumor growth *in vivo*

**Figure 6:**
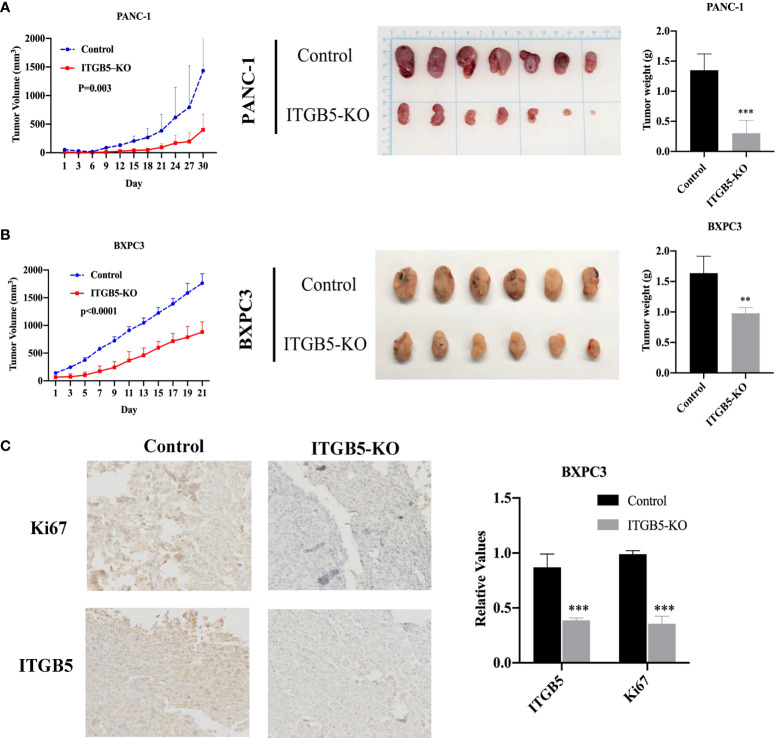
Effect of ITGB5 expression on pancreatic cancer *in vivo*. **(A, B)** Curves of tumor volume and tumor weight in ITGB5 knockout or control PANC-1 **(A)** and BXPC3 **(B)** cells. **(C)** ITGB5 and Ki67 expression in xenograft tumor tissue by IHC. **p < 0.01, ***p < 0.001.

Based on results *in vitro*, we further investigated ITGB5 function *in vivo* by constructing xenograft tumors. Considering that the knockout efficiency of sgITGB5-1 is superior to sgITGB5-2, we used ITGB5-KO constructed by sgITGB5-1 for experiment *in vivo*. ITGB5-KO and control cells were subcutaneously injected in the distal right and left lower extremities of one mouse, respectively. As shown in **Figures 6A, B**, ITGB5 depletion significantly inhibited xenograft tumor growth in both PANC-1 and BXPC3 by recording tumor volume and tumor weight. The expression of proliferating protein Ki67 in xenograft tumors was further detected by IHC, which was consistent with ITGB5 expression (**Figure 6C** **)**. When ITGB5 was downregulated, Ki67 protein expression was inhibited, which further supported the results of *in vitro* experiments that ITGB5 depletion inhibited tumor proliferation.

### ITGB5 promotes radio-resistance in PAAD

**Figure 7:**
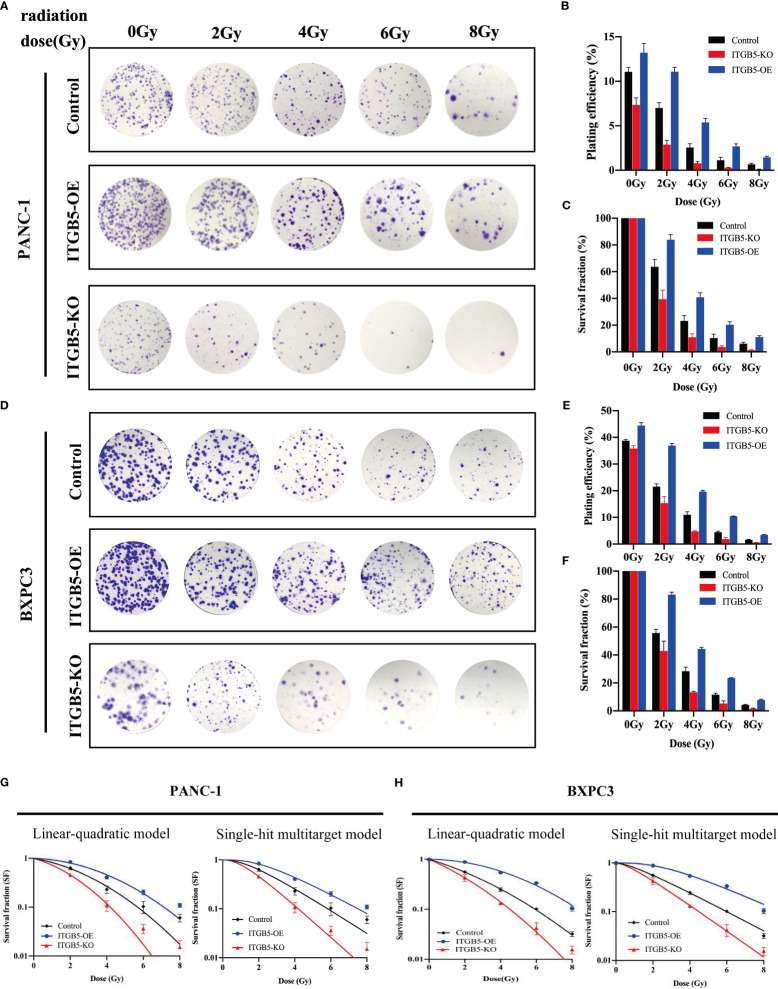
The effect of ITGB5 expression on radiation sensitization in pancreatic cancer cells. **(A, C)** The colony formation of PANC-1 **(A)** and BXPC3 **(C)** cells irradiated with different doses; **(B, E)** Plating efficiency (PE) of PANC-1 **(B)** and BXPC3 **(E)** cells; **(C, F)** Survival fraction (SF) of PANC-1 **(C)** and BXPC3 **(F)** cells; **(G-H)** Survival fraction curves of PANC-1 **(G)** and BXPC3 **(H)** cells according to Linear–quadratic model and Single-hit multitarget model. Values were presented as mean ± SD (n=3).

To investigate the influence of ITGB5 on radiation sensitivity, ITGB5-KO, ITGB5-OE, and control cells were irradiated with 0, 2, 4, 6, and 8 Gy. According to colony formation assay and survival curves generated using survival fractions at different radiation doses in **Figures 7A-F**, ITGB5 depletion inhibited the number of colonies and survival curves became steeper (**Figures 7G, H**). Sensitization enhancement ratios (SER_D0_) of ITGB5-KO and ITGB5-OE in PANC-1 cells were 1.404 and 0.862, respectively, which in BXPC3 were 1.341 and 0.864 (**Table 2**). ITGB5 overexpression in pancreatic cancer cells promoted radio-resistance.

Table 2AParameters of survival curves in PANC-1 and BXPC3 cells.

**Table:** 

Cell line	Groups	*D0* (Gy)	*N*	*Dq* (Gy)	*SF_2_* (%)	*SER_D0_*
PANC-1	Control	1.855	2.379	1.608	63.636	
	ITGB5-OE	2.152	3.395	2.630	83.838	0.862
	ITGB5-KO	1.321	2.467	1.193	45.936	1.404
BXPC3	Control	2.178	1.602	1.026	55.642	
	ITGB5-OE	2.522	3.558	3.201	88.366	0.864
	ITGB5-KO	1.624	1.617	0.781	42.831	1.341

**Table 2B:** Survival fractions (%) of PANC-1 and BXPC3 cells in different groups irradiated with different doses.

GroupDose(Gy)		Control			ITGB5-KO			ITGB5-OE		
		Average	SD	RSD	Average	SD	RSD	Average	SD	RSD
PANC-1	0	100.606	4.576	4.548	100.457	11.072	11.022	100.000	7.873	7.873
	2	63.636	5.455	8.571	45.936	2.795	6.085	83.838	3.813	4.548
	4	23.106	3.991	17.272	10.845	2.616	24.119	40.720	3.414	8.385
	6	10.303	2.922	28.364	3.653	0.791	21.651	20.202	2.314	11.456
	8	6.061	1.050	17.321	1.522	0.527	34.641	10.943	1.051	9.608
BXPC3	0	100.086	1.301	1.299	100.000	2.903	2.903	100.000	2.508	2.508
	2	55.642	2.652	4.766	42.831	7.074	16.516	88.366	0.804	0.910
	4	24.649	2.195	8.906	13.152	0.727	5.527	54.200	1.126	2.078
	6	10.140	0.241	2.375	4.214	1.177	27.941	33.423	0.225	0.674
	8	3.192	0.359	11.235	1.552	0.284	18.330	10.374	1.229	11.850

**Figure 8:**
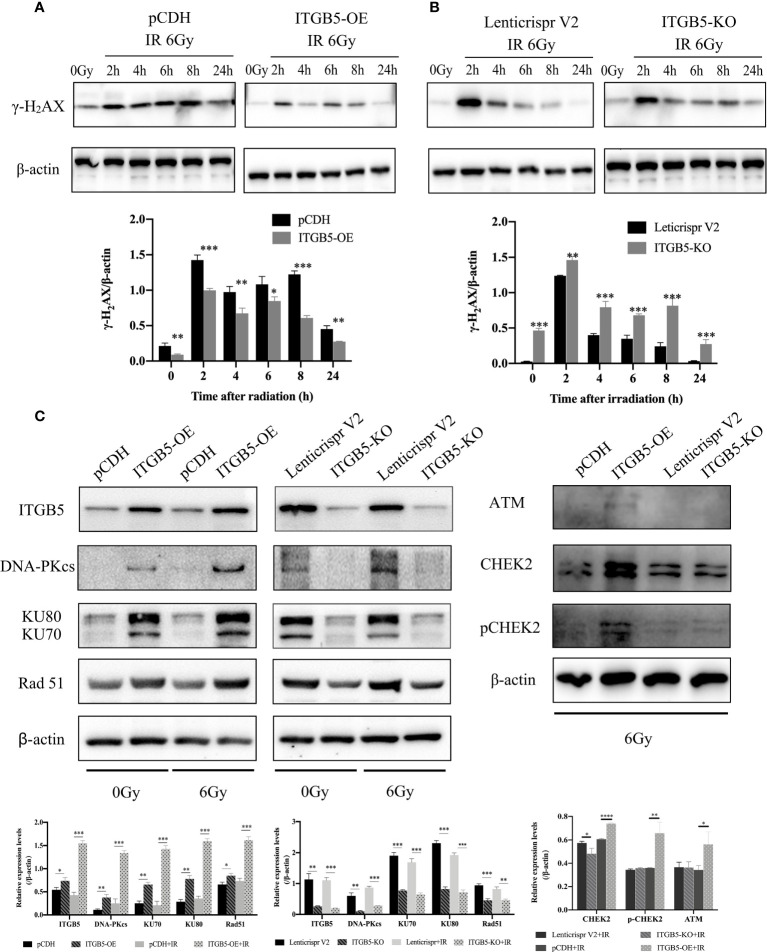
Effect of ITGB5 expression on DNA damage proteins expression levels in BXPC3 cells. **(A, B)** The effect of ITGB5 overexpression **(A)** and ITGB5 knockout **(B)** on γ-H_2_AX expression at different time points after irradiation in BXPC3 cells. **(C)** the effects of ITGB5 overexpression and knockout on DNA injury repair protein expression in BXPC3 cells before and after irradiation. *p < 0.05, **p < 0.01, ***p < 0.001, ****p < 0.0001.

We then investigated the possible mechanism by which ITGB5 induces enhanced radio-resistance in pancreatic cancer cells. Firstly, we tested whether ITGB5 overexpression induced more DNA double-strand breaks (DSBs). γ-H_2_AX, formed at DSBs damage sites, is used as a marker of DNA DSBs (26). WB was further used to detect the γ-H_2_AX protein expression of ITGB5-KO, ITGB5-OE, and control BXPC3 cells at different time points (0, 2, 4, 6, 8, 24 h) after irradiation. As shown in the results in **Figures 8A, B**, γ-H_2_AX protein expression increased 2 h after irradiation in the control group and basically dropped to normal levels after 6–8 h of irradiation. However, in the group of ITGB5-KO BXPC3 cells, γ-H2AX expression increased obviously and was still at a high expression level 24 h after irradiation; on the contrary, in the group of ITGB5-OE cells, γ-H_2_AX expression was significantly lower than that in the control group. A semiquantitative statistical analysis of the gray level of WB strips showed that 0, 2, 4, 6, 8, and 24 h after irradiation, the expression level of γ-H_2_AX in ITGB5-KO cells was significantly higher than that in the control group, and the difference was statistically significant. On the contrary, the expression level of γ-H_2_AX in ITGB5-OE cells was significantly lower than that in the control group.

Secondly, we examined DNA damage repair protein expression (**Figure 8C**). DNA-PK, ATM (ataxia telangiectasia mutated), and ATR (ataxia telangiectasia and Rad3 related) belong to the phosphatidylinositol 3-kinase-related kinase family (PI3K). DNA-PK and ATM were mainly activated by DSBs, while ATR was mainly activated by single-strand breaks (SSBs). Here, we focused on the effect of ITGB5 on the expression changes of DNA-PK and ATM targeting at DSB repair. The results showed that Rad51, DNA-PKcs, and KU70/80 protein expression levels were significantly increased in the ITGB5-OE BXPC3 cells and decreased in ITGB5-KO BXPC3 cells compared with control cells (p < 0.05). After irradiation, the expression differences of Rad51, DNA-PKcs, and KU70/80 protein between ITGB5-OE or ITGB5-KO BXPC3 cells and control cells were more obvious (p < 0.01). Another ATM-CHEK2 signaling pathway, ATM, CHEK2, and phospho-CHEK2 (pCHEK2) were highly upregulated in the group of ITGB5-OE. Abnormally increased ITGB5 promoted DNA damage repair by activating DNA-PK and ATM, which then enhanced radio-resistance.

**Figure 9:**
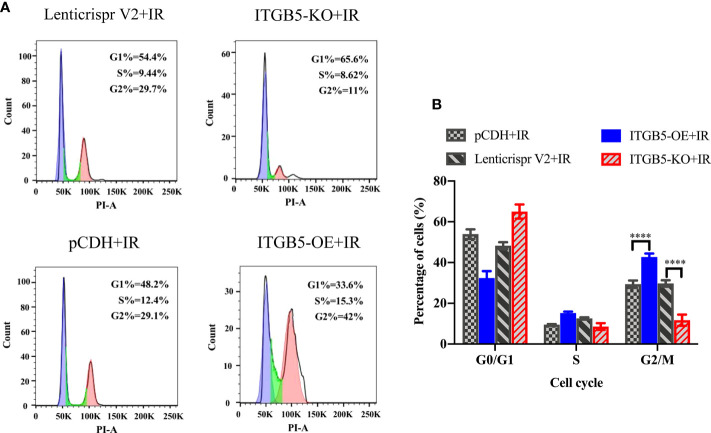
Effect of ITGB5 expression on the cell-cycle distribution in BXPC3 cells irradiated by 6 Gy. **(A)** Results of cell cycle flow cytometry in BXPC3 cells; **(B)** The statistical analysis results of cell cycle in BXPC3 cells. ****p < 0.0001.

The activation of DNA damage repair proteins may induce cell-cycle arrest. We further examined cell-cycle distribution after irradiation. As shown in **Figure 9**, in group ITGB5-OE, the proportion of G2/M phase cells was significantly increased and, adversely, the proportion of G2/M phase cells was significantly decreased in group ITGB5-KO.

**Figure 10:**
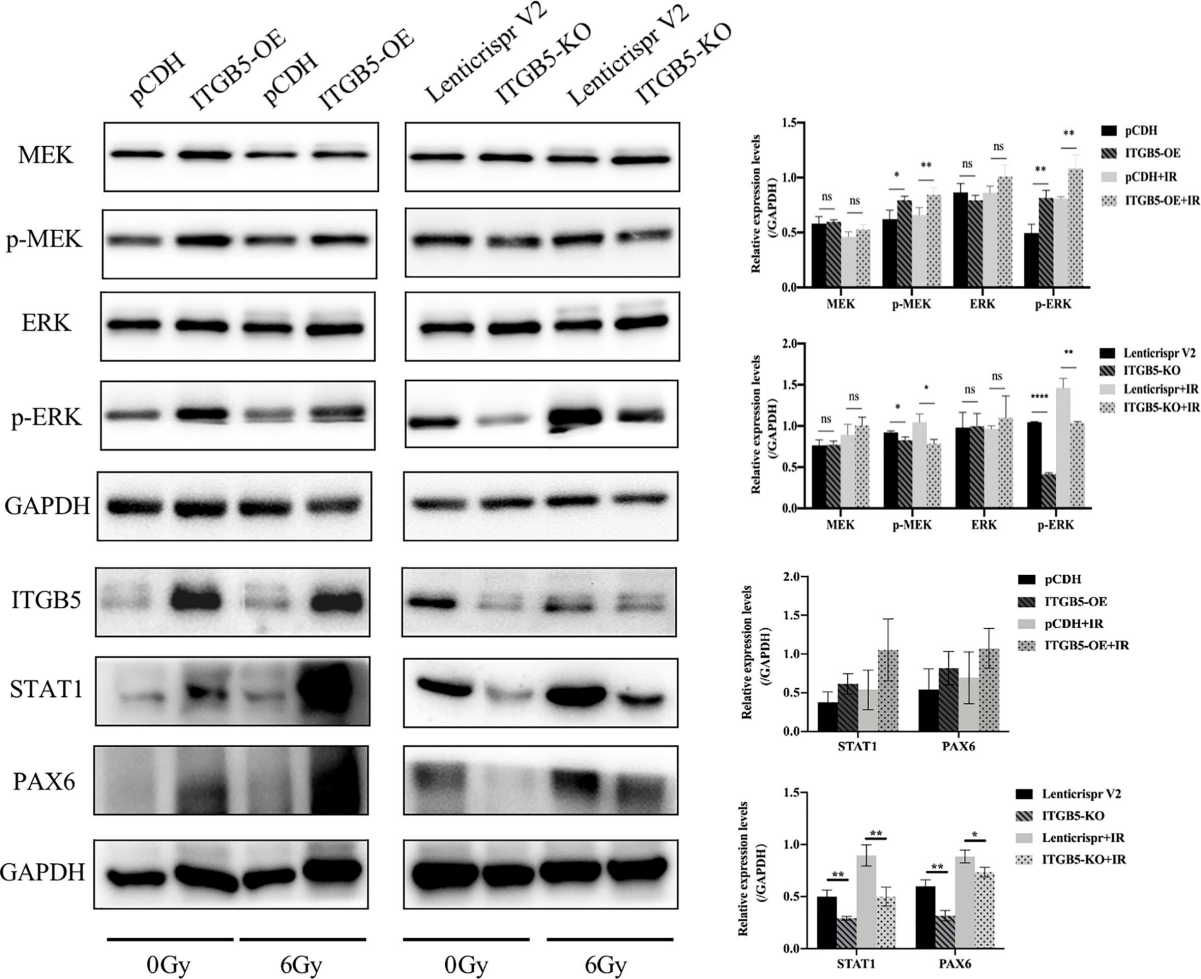
Effect of ITGB5 expression on MEK/ERK signaling protein expression levels in BXPC3 cells before and after irradiation. *p < 0.05, **p < 0.01, ****p < 0.0001. “ns”, Not Statistically.

Lastly, we examined protein expression related with the MEK/ERK signaling pathway (**Figure 10**). MEK and ERK protein expression showed no significance between ITGB5-KO or ITGB5-OE BXPC3 cells and control cells both before and after irradiation (p > 0.05). However, compared with the control cells, the protein expression levels of p-MEK and p-ERK were increased in the ITGB5-OE BXPC3 cells and significantly decreased in the ITGB5-KO BXPC3 cells, and the differences were more significant after irradiation (p < 0.05). Besides, in the ITGB5-OE group, PAX6 and STAT1 expression downstream of the MEK/ERK signaling pathway was also significantly increased before and after irradiation. These results suggested that ITGB5 promoted DNA damage repair by activating the MEK/ERK pathway and then enhanced radio-resistance.

## Discussion

Integrin, as a member of the family of cell adhesion molecules, is a bridge for intercell or cell–ECM interactions and is strongly linked to the occurrence and evolvement of multiple tumor types. Focusing on the recent findings, the important roles and related mechanisms of integrin in glioma (27–30), melanoma (31), colorectal cancer (32), and breast cancer (33) have been reported. For instance, integrin αvβ3 acts as a cancer stem cell driver factor that regulates the growth and resistance of melanoma (34, 35). The α2β1 integrin along with α3β1, α6β1, and αvβ1 integrins inhibited apoptosis of small cell lung cancer cells induced by chemotherapy and radiation (36). Integrin α2β1 has also been reported to promote tumor cell migration through epithelial-stromal transformation (EMT), leading to the dissemination process of cancers (37). Integrin-mediated cell interactions with ECM fundamentally enhanced cancer cell resistance to treatment. The study of drug-targeted integrins is of great significance for the clinical treatment of tumors, which has become a promising target for cancer treatment.

Many recent studies have reported the potential role and mechanisms of ITGB5 in tumor progression. ITGB5 mediated TGF-induced EMT in breast cancer cells (6, 38). ITGB5 promoted the occurrence of hepatocellular carcinoma by interacting with the β-catenin protein (8). Furthermore, ITGB5 has been found to be associated with immunomodulation and angiogenesis in the glioblastoma microenvironment and is required for the formation of endothelial cells into tubes (9). However, the function of ITGB5 in pancreatic cancer and its effect on radiosensitivity remain to be reported.

Our analysis of ITGB5 expression in authoritative databases and clinical samples in TMA both supported that ITGB5 is significantly upregulated in PAAD, which led to a lower probability of OS. The main causes of poor prognosis in patients with pancreatic cancer include tumor cell proliferation and metastasis. In our study, the forced expression of ITGB5 not only apparently potentiated the proliferation, migration, and invasion of pancreatic cancer cells *in vitro* but also facilitated the growth of implanted pancreatic tumors *in vivo*.

At present, RT is one of the main cancer treatment methods, and varying degrees of radio-resistance due to ionized radiation tolerance make the radiotherapy effect unsatisfactory. Consequently, it is a strategic therapeutic option to improve radiosensitivity. In our study, colony formation assay manifested that ITGB5 silencing weakened the vitality of pancreatic cancer cells after irradiation, suggesting that ITGB5 overexpression enhanced innate radiation resistance in PAAD. The primary effect of radiation on cells is to induce DSBs and trigger DDR (39), and radiation can induce large amounts of γ-H_2_AX, which can act as an early marker of post-radiation DSBs (40–43). We examined the expression of γ-H_2_AX protein at different time points after irradiation under different ITGB5 expression conditions in BXPC3 cells. We found that in ITGB5-KO BXPC3 cells, the expression level of γ-H_2_AX was significantly increased and the γ-H_2_AX peak duration was prolonged. It was also confirmed that ITGB5 knockout delayed radiation-induced DSB_S_ repair and thus improved radiosensitivity.

Subsequently, we attempted to further validate the potential molecular mechanism of ITGB5 in decreasing radiosensitivity. Irradiation can induce a series of DDRs; cell-cycle arrest and DNA damage repair are the two main forms of DDRs that protect cancer cells from irradiation (44). Moreover, in mammals, non-homologous end joining (NHEJ) and homologous recombination (HR) are two major DDRs (45). Three kinases in the PI3K family play a leading role in DDR: DNA-PK, ATM, and ATR (44). DNA-PK and ATM mainly responded to DSBs, while ATR mainly responded to SSBs (46, 47). In addition, DNA-PK mainly participates in NHEJ and ATM mainly participates in HR (46). We hypothesized that targeting ITGB5 could affect pancreatic cancer radiosensitivity through the DNA damage repair pathway. Moreover, in our data, high expression of ITGB5 not only induced G2/M phase cell arrest but also effectively activated DNA-PK and ATM-related DNA damage repair signaling pathways. These results suggested that ITGB5 increased the radiation resistance of pancreatic cancer cells by promoting DSB damage repair.

Previous studies demonstrated that the abnormal activation of Ras/Raf/MEK/ERK cascade mediates resistance to irradiation in cancer cells (48). Huang et al. (49) reported that the ERK pathway can increase radiation resistance of glioma. Another study found that sorafenib increased radiosensitivity of lung cancer cells by inhibiting ERK phosphatization (50). Our study demonstrated that the silencing of ITGB5 leads to downregulation of phosphorylated MEK/ERK, which enhanced DNA damage and radiosensitivity, which is in line with previous studies. Besides, activation of the ERK pathway can further promote DSB-induced ATM activation (51, 52).

On the whole, our experiment results unveil ITGB5 as a novel anticancer target and a potential prognostic marker in PAAD.

## Data availability statement

The original contributions presented in the study are included in the article/supplementary material. Further inquiries can be directed to the corresponding authors.

## Ethics statement

Ethical review and approval was not required for the study on human participants in accordance with the local legislation and institutional requirements. Written informed consent for participation was not required for this study in accordance with the national legislation and the institutional requirements. The animal study was reviewed and approved by Xuzhou Medical University.

## Author contributions

XW performed the study design, wrote and edited the manuscript. SC performed the experimental research. XC performed data analysis and conducted part experiments in revised manuscript. HQ contributed to the writing and editing of the manuscript. WW, NZ, WL, TW contributed to data analysis. XD and LZ performed the study design, paper guidance, and financial support. All authors contributed to the article and approved the submitted version.

## Funding

This work was supported by National Natural Science Foundation of China (No. 81972845), and Specialist Team in Clinical Medicine of Xuzhou (No.2019TD003)

## Conflict of interest

The authors declare that the research was conducted in the absence of any commercial or financial relationships that could be construed as a potential conflict of interest.

## Publisher’s note

All claims expressed in this article are solely those of the authors and do not necessarily represent those of their affiliated organizations, or those of the publisher, the editors and the reviewers. Any product that may be evaluated in this article, or claim that may be made by its manufacturer, is not guaranteed or endorsed by the publisher.
