# Corrigendum: ITGB5 promotes innate radiation resistance in pancreatic adenocarcinoma by promoting DNA damage repair and the MEK/ERK signaling pathway

**DOI:** 10.3389/fonc.2024.1381151

**Published:** 2024-02-22

**Authors:** Xin Wen, Si Chen, Xueting Chen, Hui Qiu, Wei Wang, Nie Zhang, Wanming Liu, Tingting Wang, Xin Ding, Longzhen Zhang

**Affiliations:** ^1^ Department of Radiation Oncology, Affiliated Hospital of Xuzhou Medical University, Xuzhou, China; ^2^ Cancer Institute of Xuzhou Medical University, Xuzhou, China; ^3^ Department of Radiation Oncology, The Second Affiliated Hospital of Xuzhou Medical University, Xuzhou, China; ^4^ Jiangsu Center for the Collaboration and Innovation of Cancer Biotherapy, Xuzhou, China

**Keywords:** pancreatic adenocarcinoma (PAAD), ITGB5, radio-sensitivity, MEK/ERK signaling pathway, DNA damage repair

**Error in Figure/Table**

In the published article, there was an error in **Figure 5** and **Figure 7** as published. Due to our careless work, we took multiple images of each group at the same time and saved all the images in the same folder. In **Figure 5**, images in pCDH were taken following taking images in sg-ITGB5, so that two images are confused partly. Similar mistake happened in **Figure 7**.]. The corrected [**Figure 5** and **Figure 7**] and its caption [**Figure 5** The effect of ITGB5 expression on migration and invasion on pancreatic cancer cells. **p<0.01, ***p<0.001] and [**Figure 7** The effect of ITGB5 expression on radiation sensitization in pancreatic cancer cells. (A, C) The colony formation of PANC-1 (A) and BXPC3 (C) cells irradiated with different doses; (B, E) Plating efficiency (PE) of PANC-1 (B) and BXPC3 (E) cells; (C, F) Survival fraction (SF) of PANC-1 (C) and BXPC3 (F) cells; (G-H) Survival fraction curves of PANC-1 (G) and BXPC3 (H) cells according to Linear-quadratic model and Single-hit multitarget model. Values were presented as mean ± SD (n=3).] appear below.

**Figure 5:**
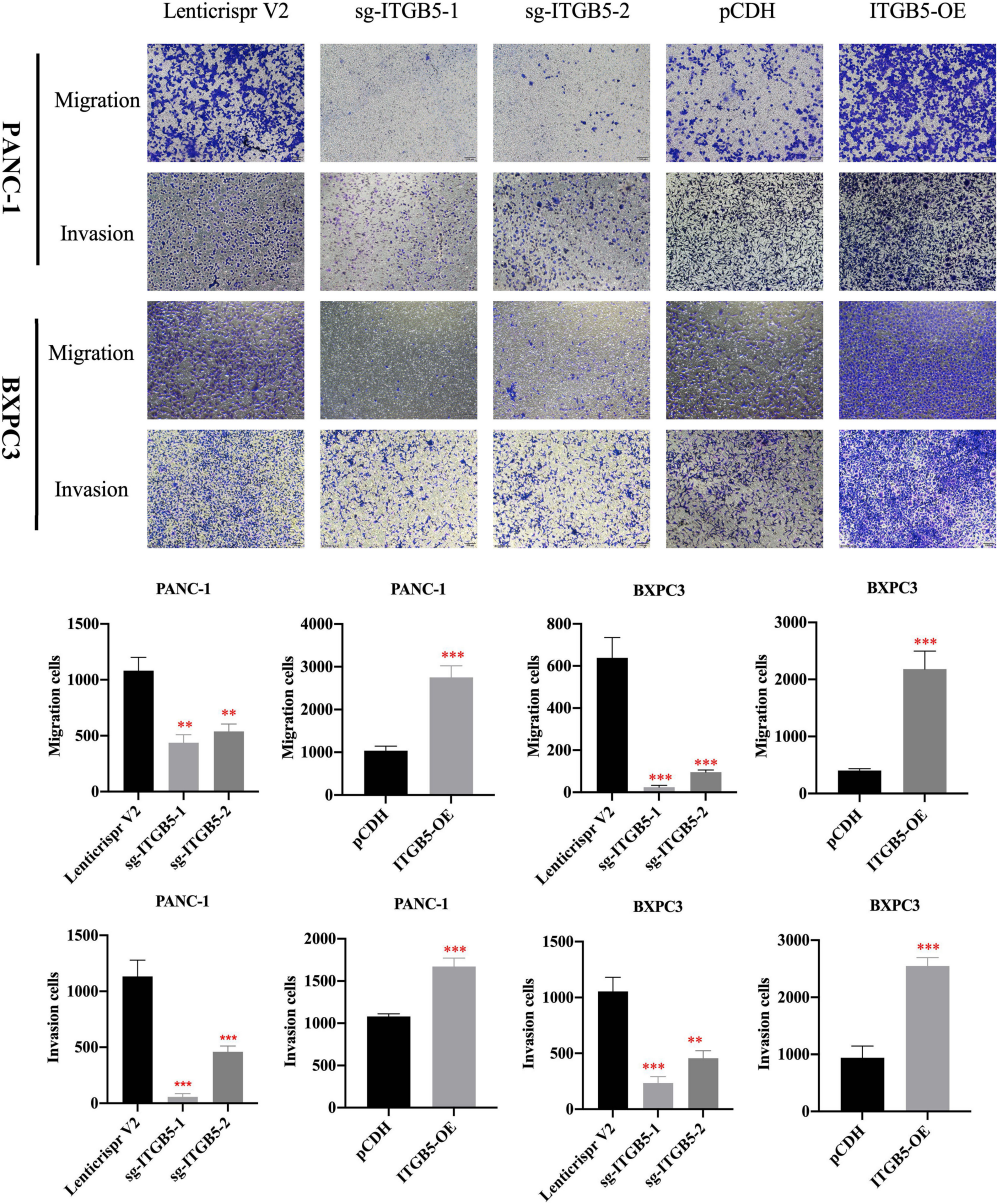
The effect of ITGB5 expression on migration and invasion on pancreatic cancer cells. **p<0.01, ***p<0.001.

**Figure 7:**
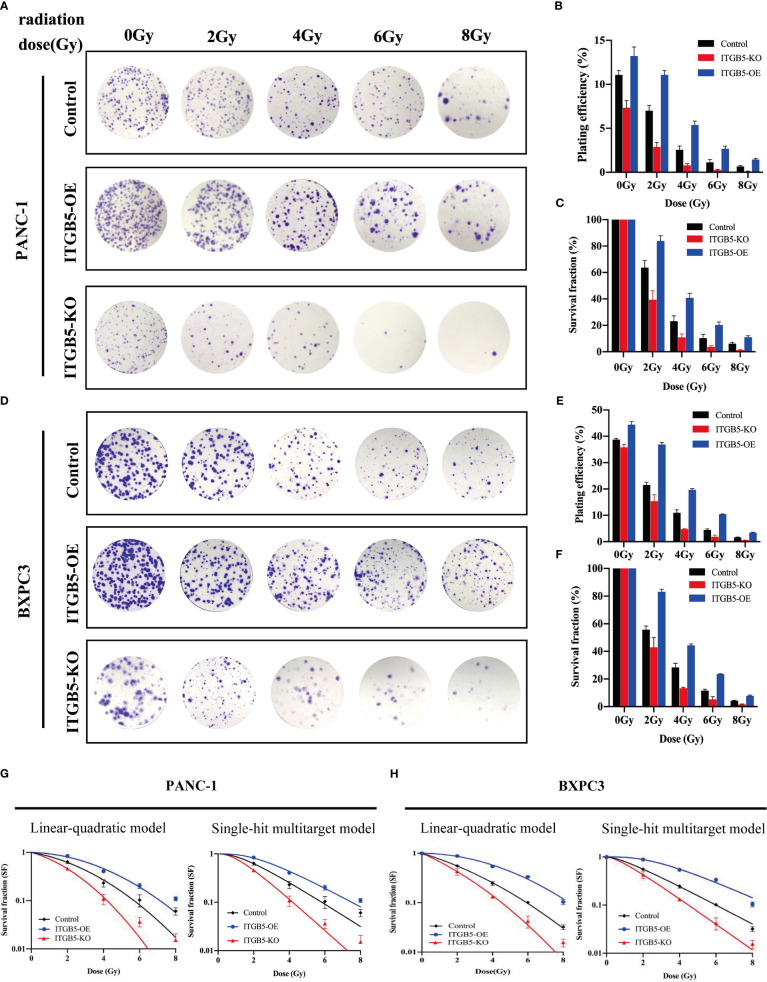
The effect of ITGB5 expression on radiation sensitization inpancreatic cancer cells. **(A, C)** The colony formation of PANC-1 **(A)** and BXPC3 **(C)** cells irradiated with different doses; **(B, E)** Plating efficiency (PE) of PANC-1 **(B)** and BXPC3 **(E)** cells; **(C, F)** Survival fraction (SF) of PANC-1 **(C)** and BXPC3 **(F)** cells; **(G-H)** Survival fraction curves of PANC-1 **(G)** and BXPC3 **(H)** cells according to Linear-quadratic model and Single-hit multitarget model. Values were presented as mean ± SD (n=3).

The authors apologize for this error and state that this does not change the scientific conclusions of the article in any way. The original article has been updated.

